# The Effect of Flexor Hallucis Longus Morphology on Os Trigonum Syndrome

**DOI:** 10.1177/23259671261422731

**Published:** 2026-04-16

**Authors:** Nathan Chaclas, Morgan E. Swanson, Vandan Patel, Jie C. Nguyen, Kathleen J. Maguire

**Affiliations:** * University of Pennsylvania Perelman School of Medicine, Philadelphia, Pennsylvania, USA; † Department of Orthopaedics, The Children's Hospital of Philadelphia, Philadelphia, Pennsylvania, USA; ‡ Department of Radiology, The Children's Hospital of Philadelphia, Philadelphia, Pennsylvania, USA; Investigation performed at The Children's Hospital of Philadelphia, Philadelphia, Pennsylvania, USA

**Keywords:** ankle, flexor hallucis longus, os trigonum, pediatric sports medicine, posterior ankle impingement

## Abstract

**Background:**

Os trigonum (OT) is an accessory bone at the posterior ankle that can lead to posterior ankle impingement. The flexor hallucis longus (FHL) muscle is a potential contributor to this pathology as it passes through the fibro-osseous tunnel behind the medial malleolus adjacent to the talus.

**Purpose:**

To compare the morphology of the FHL muscle belly between patients with symptomatic and asymptomatic OT.

**Study Design:**

Cross-sectional study; Level of evidence, 3.

**Methods:**

We performed a retrospective analysis of pediatric patients with an OT who underwent ankle magnetic resonance imaging at a single center from 2015 to 2023. We collected descriptive data, imaging indications, and the following radiographic measurements: distance from the distal end of the tibia to the FHL muscle musculotendinous junction (MTJ) (absolute FHL MTJ distance); ratio of absolute MTJ distance to the distance from the tibia to the tarsal tunnel (relative FHL MTJ distance); axial area of the FHL muscle belly or tendon at the midpoint of the OT/Stieda process; and axial and sagittal areas of the OT. A 1-tailed independent *t* test was used to compare continuous variables, guided by the Levene test for equality of variances.

**Results:**

The FHL was positioned significantly lower in the symptomatic group (n = 14) than in the asymptomatic group (n = 21), when represented by both absolute (*P* = .044) and relative FHL MTJ distances (*P* = .037). The relationship between symptoms and sex was significant (*P* = .024). The relationships were also significant between symptoms FHL MTJ distance/patient height (*P* = .049) and FHL MTJ distance/patient body mass index (*P* = .041). There was no significant difference in age, FHL axial area, or OT length, area, and volume between groups (*P* > .05).

**Conclusion:**

These findings suggest that the FHL muscle may play a role in the pathogenesis and clinical presentation of OT syndrome. We propose that the evaluation and treatment of OT syndrome should include the assessment and management of the FHL muscle, and that future studies should explore the mechanisms underlying the association between the FHL muscle and OT syndrome.

An os trigonum (OT) is an accessory ossicle located at the posterolateral aspect of the talus, adjacent to the lateral tubercle of the posterior process of the talus.^9^ It is a normal anatomical variant that results from the failure of fusion of the secondary ossification center of the lateral tubercle with the body of the talus during childhood^13^ (Figure 1). The prevalence of OT ranges from 1% to 30% in the general population, and it is usually asymptomatic and incidentally identified.^4,15^ However, in some cases, OT can cause posterior ankle impingement syndrome, also known as OT syndrome, which is characterized by pain, swelling, and reduced plantarflexion of the ankle.^9^ This overuse condition often comes about due to activities that require repetitive ankle plantarflexion. Stieda process (SP)—another normal anatomic variant resulting from elongation of the lateral tubercle of the posterior process of the talus—can also cause posterior ankle impingement syndrome. The treatment of OT syndrome depends on the severity and duration of the symptoms. This can range from conservative measures such as rest, ice, anti-inflammatory medications, physical therapy, and corticosteroid injections, to surgical interventions, such as arthroscopic or open excision of the OT.^13^

**Figure 1.**
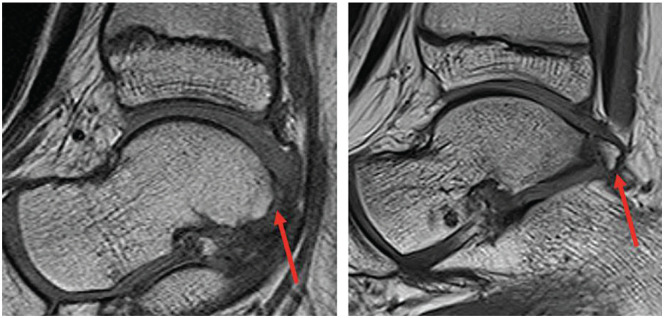
An OT is visualized in the sagittal T1-weighted MRI on the right, compared with a similar slice from an ankle MRI of a patient without an os trigonum (left). MRI, magnetic resonance image; OT, os trigonum.

The flexor hallucis longus (FHL) is one of the deep muscles of the posterior compartment of the leg, and it originates from the posterior surface of the fibula, the interosseous membrane, and the adjacent fascia.^8^ It passes through the fibro-osseous tunnel posterior to the medial malleolus, where it is enveloped by a synovial sheath, and then runs along the medial side of the plantar foot, crossing the sustentaculum tali of the calcaneus and the plantar surface of the navicular.^8^

Several studies have investigated the anatomical and morphological characteristics of the FHL muscle and OT in relation to OT syndrome. Various imaging modalities—such as radiography and magnetic resonance imaging (MRI)—have been employed.^3,6,12^ There remains a need for a comprehensive and comparative study of the FHL muscle and the OT in patients with symptomatic versus asymptomatic OT. We propose a standardized MRI study that provides excellent soft-tissue differentiation to analyze the relationship between the anatomy and morphology of the FHL muscle belly and the OT.

This study aimed to compare the anatomic position and morphology of the FHL muscle belly between patients with symptomatic and asymptomatic OT. We hypothesized that the FHL muscle belly would be lower lying in patients with symptomatic OT than in patients with asymptomatic OT. We also hypothesized that the FHL muscle belly would be larger in cross-sectional area at the OT level in symptomatic patients.

## Methods

After approval from our center's institutional review board, we performed a retrospective analysis of MRI scans of the ankle identified via a billing query for OT using the International Classification of Diseases, 10th Revision (ICD 10 Q68.8) and ankle MRI (Current Procedural Terminology [CPT] 73721, 72723). Patients were included based on the presence of OT or SP (hereafter grouped together) on MRI. Patients were excluded based on confounding comorbidities such as neuromuscular or syndromic conditions affecting lower extremity anatomy, as well as a history of previous ankle surgery or trauma. Patients were further excluded if they had other ankle or foot pathologies that disrupted the necessary landmarks for study measurements, such as fractures, dislocations, infections, tumors, or congenital anomalies.

Patients were divided into symptomatic and asymptomatic OT cohorts via the presence of a diagnosis of posterior ankle impingement syndrome by an attending orthopaedic surgeon or sports medicine physician (K.M.). Descriptive data—including age and sex—were abstracted from the electronic medical record.

All participants underwent MRI examinations of the ankle on either a 3-T magnet (Magnetom Prisma; Siemens Healthineers) or a 1.5-T magnet (Avanto; Siemens Healthineers) using a 16-channel foot/ankle surface coil (Siemens Healthineers). Images were obtained with the patient in a supine position and included axial and sagittal proton density-weighted (repetition time/echo time [TR/TE] = 3000/30–40 ms), axial and coronal T2-weighted (TR/TE = 3500–5000/65–80 ms), and coronal T1-weighted (TR/TE = 550–700/10–12 ms) sequences. The field of view ranged between 12 and 14 cm (dependent on the patient's size), the matrix size ranged between 256 and 384, and the section thickness was 3 mm with an interslice gap ranging^10^ from 0% to 20%.

Three orthopaedic researchers (N.C., M.S., V.P.), each with at least one year of orthopedic research experience, independently reviewed the OT/SP patient MRIs. MRI measurements used to assess the FHL musculotendinous junction (MTJ) location are described in Table 1. To normalize the MTJ distance across body sizes, we divided the absolute MTJ distance by the following denominators: the tibia-to-tarsal tunnel distance (yielding a relative MTJ distance), patient height, and body mass index. This allows us to relativize the MTJ distance using several pertinent body habitus metrics to better appreciate the data we gather from our study cohort. MRI measurements are further detailed in Table 1. OT/SP volume was reconstituted using sagittal length in the axial plane multiplied by sagittal cross-sectional area.

**Table 1:** Description of MRI Measurements*^*a*^*

Measurement	Slice Used	Description of Measurement		Image
Absolute MTJ distance	Mid-sagittal slice with the largest cross-section of the distal tibia	Distance from the posteroinferior corner of the distal tibia to the MTJ	Parallel to the tibial shaft. If the MTJ was proximal to the distal edge of the tibia, the value is negative	Figure 2
Tibia to tarsal tunnel	Mid-sagittal slice with the largest cross-section of the FHL tendon	Distance from the posteroinferior corner of the distal tibia to the curvature point at the subtalar joint	Parallel to the tibial shaft	Figure 3
OT length	Mid-sagittal slice with the largest cross-section of OT/SP	Distance from the most anterior point to the most posterior point of the OT/SP	Parallel to the long axis of the OT/SP	Figure 4
OT area	Mid-sagittal slice with the largest cross-section of OT/SP	Cross-sectional area of the OT/SP	Includes cartilaginous edges	Figure 4
FHL cross-sectional area	Axial slice at the midpoint of OT or SP	Cross-sectional area of the FHL muscle and/or tendon	If no muscle belly fibers were present, the cross-sectional area of the FHL tendon alone was measured	Figure 5

a FHL, flexor hallucis longus; MTJ, musculotendinous junction; Os, ossicle; OT, Os trigonum; SP, Stieda process.

**Figure 2.**
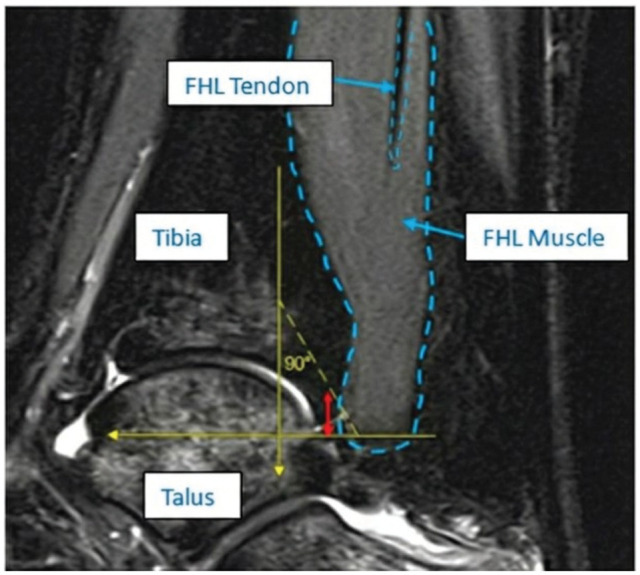
Mid-sagittal T1-weighted MRI shows a 2-mm absolute MTJ distance (red arrow) on the right ankle of a 15-year-old female with os trigonum syndrome. Note that the label "FHL Tendon" points to the intramuscular portion of the tendon extending proximally within the muscle belly. The absolute MTJ distance is measured by the red arrow, representing the vertical distance starting from the level of the posteroinferior corner of the distal tibia (indicated by the horizontal yellow reference line) up to the distal-most point of the MTJ, parallel to the tibial shaft (vertical, yellow line). FHL, flexor hallucis longus; MRI, magnetic resonance imaging; MTJ, musculotendinous junction.

**Figure 3.**
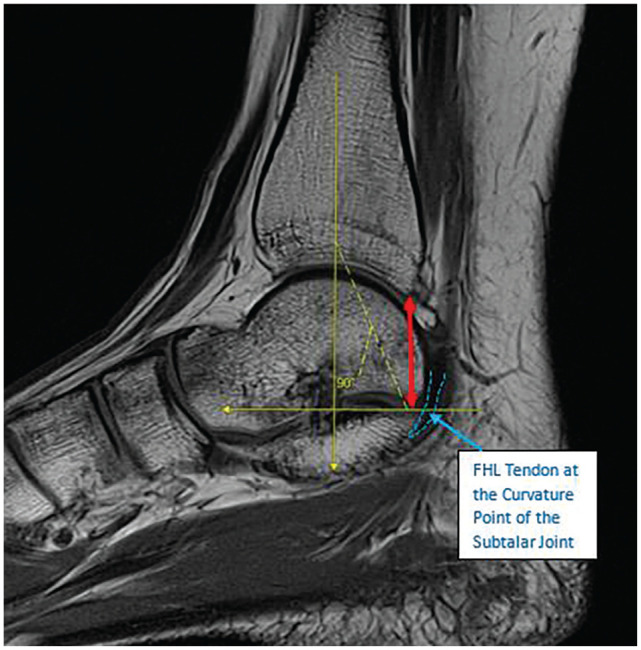
Mid-sagittal PD-weighted MRI shows a 22.5-mm tibia to tarsal tunnel measurement (red arrow) on the right ankle of a 15-year-old female with OT syndrome. The distance from the posteroinferior corner of the distal tibia to the curvature point at the subtalar joint (horizontal yellow line) is measured parallel to the tibial shaft (vertical yellow line). FHL, flexor hallucis longus; MRI, magnetic resonance imaging; OT, os trigonum; PD, proton density.

**Figure 4.**
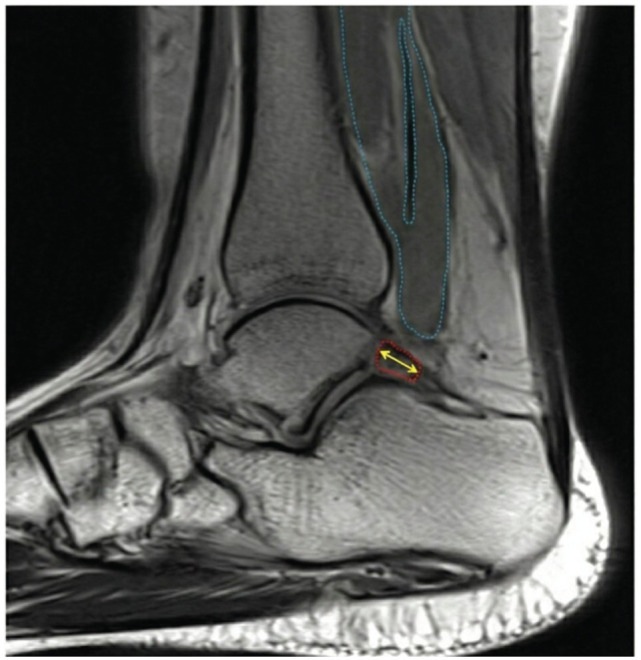
Mid-sagittal T1-weighted image shows a 38.9-mm^2^ OT area measurement (red outline) and the OT length measurement (yellow arrow) on the left ankle of a 16-year-old female with OT syndrome. The FHL muscle belly and tendon are also visualized and outlined in blue. FHL, flexor hallucis longus; OT, os trigonum.

**Figure 5.**
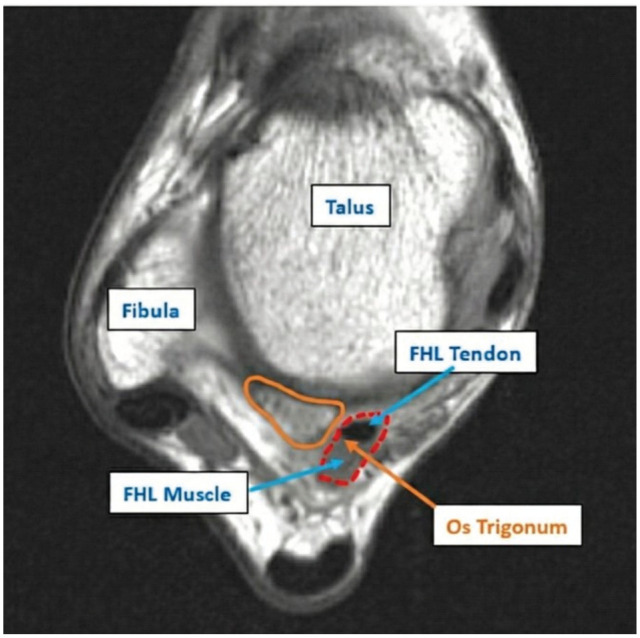
A 35.6-mm^2^ FHL. The cross-sectional area (red outline) measurement is shown on the left ankle of a 9-year-old female with an asymptomatic OT. The OT is also outlined in orange and labeled. The axial slice at the midpoint of the os trigonum was used. FHL, flexor hallucis longus; OT, os trigonum.

Interobserver agreement among the researchers was assessed using the intraclass correlation coefficient (ICC) for continuous variables. The ICC was categorized^1^ as poor (<0.5), moderate (0.5 to <0.75), good (0.75 to <0.9), and excellent (0.9 to 1). A 1-tailed independent *t* test was used to compare continuous variables between the symptomatic and asymptomatic subcohorts, guided by the Levene test for equality of variances. All analyses were performed using SPSS Version 29.

## Results

Fourteen patients with symptomatic OT or SP and 21 patients with asymptomatic OT or SP who underwent MRI between 2015 and 2023 were identified. The mean ICC ranged from good to excellent (0.706-0.957) across all measurements. Interrater reliability results are described in Table 2.

**Table 2:** ICC Results*^*a*^*

Measurement	ICC
Tibia to tarsal tunnel	0.957
FHL MTJ distance	0.935
FHL area	0.812
OT/SP area	0.899
OT/SP length	0.706

a FHL, flexor hallucis longus; ICC, intraclass correlation coefficient; MTJ, musculotendinous junction; OT, Os trigonum; SP, Stieda process.

The FHL muscle belly was significantly lower lying in the symptomatic subcohort of patients with OT than in the asymptomatic subcohort (17 ± 7.3 mm vs 11 ± 10 mm), when represented by both absolute (*P* = .044) and relative FHL MTJ distances (*P* = .037). There was no significant difference between the cross-sectional sagittal area of the OT (*P* = .097), the sagittal length in the axial plane (*P* = .244), or the reconstituted OT volume (*P* = .075) between the 2 groups. Further, the cross-sectional axial area of the FHL did not differ between the 2 groups (*P* = .530). Imaging characteristics are further detailed in Table 3.

**Table 3:** OT/SP Subcohort Stratified Characteristics and MRI Indices*^*a*^*

Variable	Asymptomatic OT/SP N = 21	Symptomatic OT/SP N = 14	*P*
Age, years	13.9 ± 2.4	13.6 ± 2.1	.913
Sex			
Male	9 (39)	1 (6)	**.024**
Female	14 (61)	16 (94)	
BMI, kg/m^2^	23.2 ± 5.6	22.7 ± 5.2	.674
Absolute FHL MTJ distance, mm	11.8 ± 9.7	15.3 ± 8.6	**.044**
MTJ distance/patient height	0.1 ± 0.1	0.1 ± 0	**.049**
MTJ distance/BMI	0.5 ± 0.5	0.8 ± 0.4	**.041**
Relative FHL MTJ distance	0.5 ± 0.5	0.7 ± 0.4	**.037**
FHL cross-sectional area, mm^2^	25.4 ± 21.3	29.4 ± 22.3	.530
OT/SP area, sagittal, mm^2^	51.3 ± 29	37 ± 20.4	.097
OT/SP length, axial, mm	10 ± 3.2	8.9 ± 2	.244
OT/SP volume, reconstituted, mm^3^	276 ± 175	200 ± 93	.075

a Data are presented as mean ± SD or n (%). Bold *P* values indicate statistical significance (*P* < .05). BMI, body mass index; FHL, flexor hallucis longus; MTJ, musculotendinous junction; OT, Os trigonum; SP, Stieda process.

## Discussion

This study demonstrates that the FHL muscle belly is lower lying in symptomatic patients than in asymptomatic patients with OT. This finding suggests that distal extension of the FHL muscle belly may contribute to the development of OT syndrome due to overstuffing the posteromedial ankle. Specifically, the presence of muscle belly tissue within the fibro-osseous tunnel, a space typically occupied by the thinner tendon, may increase the volume within the canal regardless of whether the muscle belly itself is hypertrophied. Previous studies have reported that the FHL muscle can be hypertrophied or inflamed in these patients, and that concomitant FHL tenolysis or debridement of the surrounding synovitis during OT excision may improve symptoms.^5,14^ Our study provides further evidence that the FHL muscle belly can be involved in the pathophysiology of OT syndrome and may be a potential target for treatment.

The FHL muscle is a potential contributor to the development and symptoms of OT syndrome, as it can place mechanical stress on the OT and the adjacent structures during ankle plantarflexion and great toe flexion.^2,11^ Moreover, the FHL muscle can be affected by the presence of OT, as it can cause compression, irritation, or inflammation of the FHL tendon and its synovial sheath, leading to the common finding of FHL tenosynovitis in patients with OT syndrome.^2,11^ FHL tenosynovitis can manifest as pain, swelling, and crepitus along the course of the FHL tendon, particularly posterior to the medial malleolus and under the sustentaculum tali. FHL tenosynovitis can also impair FHL function, resulting in reduced strength and endurance for great toe flexion and plantarflexion.^2,11^ Therefore, the FHL muscle and the OT have a complex and dynamic relationship, which can influence the pathogenesis and clinical presentation of OT syndrome.^2,11^ Our study results suggest that compressive pathology leading to posterior ankle impingement can be multifactorial: an OT alone may not be enough to cause pathology, but can become symptomatic in patients with a relatively distal FHL muscle belly. This muscle belly may, in turn, become symptomatic from the compression and friction in the region. While this anatomical predisposition is static, conservative treatment remains effective in many cases by addressing the acute secondary inflammation and synovitis that result from the mechanical impingement. Addressing posterior ankle impingement syndrome should be multifaceted in select cases, as patients may stand to benefit from treatment tailored to their individual contributing factors.^9,7^

Our study has some limitations, largely due to its retrospective design and the limited MRI measurements, which reduced our sample size. The cross-sectional design limited our ability to assess outcomes and long-term follow-up for our study cohort. Further, while our study cohort was a heterogeneous mix of patients at a large urban tertiary care site, results were abstracted from a single center, limiting the generalizability of our study findings. Additionally, although the reviewers were blinded to the diagnosis at the time of MRI review, they were not blinded to any MRI findings that could have supported the diagnosis of symptomatic OT. These could include edema around the fibers of the FHL tendon or partial tears of the FHL tendon near the OT, visible on MRI. Moreover, we did not include data on the patients' activity level or type of activity. Consequently, we could not assess whether specific high-demand activities, such as dance, contribute to adaptive muscle hypertrophy that might influence the position of the musculotendinous junction in symptomatic patients. Finally, the electronic medical record limited our functional and activity assessment to what was contained within the treatment team documentation. Future studies should include larger and more diverse populations, longitudinal follow-up, and objective measures of ankle function and FHL muscle activity.

## Conclusion

This study reinforces the concept that FHL morphology may play a role in the symptomatic presentation of OT syndrome. Given that this ossicle is present in a large portion of the population, it is important to understand the factors that contribute to symptom development. Providers can use this information when evaluating and treating posterior ankle impingement.
